# Peripheral T cell lymphoma coexisting with Castleman's disease: A case report and literature review

**DOI:** 10.1097/MD.0000000000018650

**Published:** 2020-01-10

**Authors:** Shuyan Liu, Yuanwen Wang, Tonglin Hu, Chunli Zhang, Zhiyin Zheng

**Affiliations:** a Department of Hematology, The First Affiliated Hospital of Zhejiang Chinese Medical University, Zhejiang Provincial Hospital of Traditional Chinese Medicine; b First Clinical Medicine College, Zhejiang Chinese Medical University; c Department of Pathology, The First Affiliated Hospital of Zhejiang Chinese Medical University, Zhejiang Provincial Hospital of Traditional Chinese Medicine, Hangzhou, Zhejiang Province, China.

**Keywords:** Castleman disease, chidamide, peripheral T cell lymphoma

## Abstract

**Rationale:**

Peripheral T cell lymphoma, coexisting with Castleman's disease (CD), is rarely seen in clinical practice and is not frequently reported in the literature.

**Patient concerns:**

A 68-year-old female was admitted to our hospital for the first time due to “multiple lumps in the neck that progressively enlarged over 7 months”. 1.5 years later, the patient returned to our hospital complaining of “ difficulty breathing and purulent blood in the mouth for more than 20 days”.

**Diagnosis:**

The postoperative pathology from the (right) cervical lymph node biopsy confirmed the diagnosis of Castleman Disease (Vascular follicular type). 1.5 years after the diagnosis of CD, the patient developed secondary peripheral T cell lymphoma of unspecified type (PTCL-U).

**Interventions:**

The patient received 5 courses of chemotherapy: 2 courses of CHOP, Chidamide combined with GemOx, GDP and Hyper CVAD Bregimen.

**Outcomes:**

After 3 courses of treatment, the curative effect was partly remitted (PR). The patient was discharged in a good condition and the follow-up was uneventful.

**Lessons:**

The mechanism responsible for CD concurrent or secondary lymphoma is not clear. Epstein-Barr virus (EBV) infection may be the most common reason of CD and PTCL-U. Further understanding the mechanisms of the condition is needed.

## 1 Introduction

Castleman's Disease is a kind of pathological change characterized by chronic lymphatic tissue reaction hyperplasia, which is uncommon in the clinic. Relevant statistical data showed that the incidence of CD is 21 to 25 cases per million.^[1]^ CD lacks specific clinical symptoms, and the diagnosis is mainly based on certain histopathological features and the immunohistochemistry; the prognosis is related to the type of pathology. Localized CD can be completely excised by surgical resection. Multicentric CD is always involved in multiple systems, and there is no standard effective treatment. For this reason, the prognosis is poor; the average survival time is just 27 months. Besides that, often secondary Kaposi sarcoma (KS), Hodgkin's lymphoma (HL), non-Hodgkin's lymphoma (NHL), Plasmacytoma and other malignancies may develop.^[2]^ We searched medical journal databases in PubMed using the key words: "Castleman disease" and "Lymphoma" from 1988 to 2018, and collected almost 120 published cases of CD that coexisted with lymphomas. From these reports, the majority of CD cases were transformed into B-cell lymphomas, whilst secondary T-cell lymphomas accounted for less than 5%. Only one definitive diagnosis, PTCL-U secondary to CD, was reported in nearly 20 years.^[3]^

## 2 Patient consent

The patient provided informed consent for publication of the case. This case report was approved by the ethical committee of The First Affiliated Hospital of Zhejiang Chinese Medical University.

## 3 Case report

**Figure 1:**
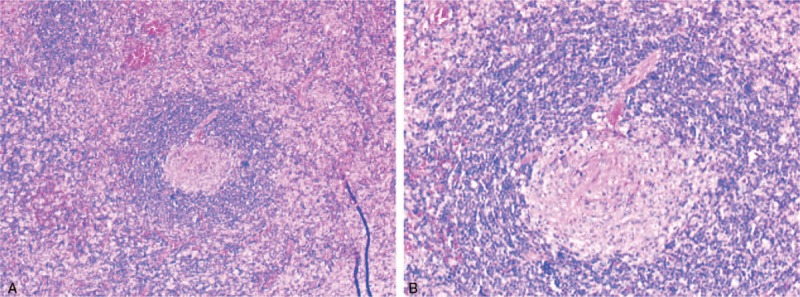
Glass-denatured blood vessels can be seen in the follicular germinal center, vascular endothelial cell hyperplasia is obvious, with “lollipop-like” characteristics. A (HE × 40); B (HE × 100).

A 68-year-old female was admitted to our hospital for the first time on Nov 4th, 2015 due to multiple lumps in her neck that progressively enlarged within 7 months. Physical examination revealed multiple palpable swollen lymph nodes on both sides of the neck, ranging in size from beans to walnuts. The masses were soft and smooth, but were not fused; furthermore, there was no apparent tenderness. Other superficial lymph nodes were not significantly enlarged. The cardiopulmonary system was unremarkable, and neither the liver nor spleen was enlarged. The neurological examination was negative. Relevant indicators revealed: WBC 5.3 × 10^9^/L, Hb 141 g/L, PLT 206 × 10^9^/L, LDH 211U/L, EBV-DNA 7.0E3/ml, negative HIV+HBV, and tumor relevant tests were negative. B-ultrasound revealed bilateral multiple enlarged lymph nodes in the neck; largest masses on the right side was approximately 2.78 × 1.55 cm, and 2.5 × 1.9 cm on the right side. Multiple inguinal lymph nodes were enlarged, the largest on the right side was about 2.4 × 0.6 cm, and 2.4 × 0.6 cm on the left side. Abdominal B-ultrasound did not show any enlarged lymph nodes. Chest CT plain scan revealed that the mediastinal lymph nodes were not enlarged, and the lung had no obvious abnormalities. On Nov 10th, 2015, the patient underwent a right cervical lymph node biopsy under local anesthesia. The postoperative pathology showed: lymphoid follicular hyperplasia, partial growth center dilatation, vitreous degeneration of blood vessels in some follicular germinal centers, interstitial vascular proliferation, and scattered or focal plasma cell infiltration. Immunohistochemically tests showed: CD20(+), CD79a(+), Bcl-2(+), CD3(+), CD5(+), CD45RO(+), CyclinD1(−), CD10(−), MUM1(scattered or focal +), P53(−), Ki-67(5%+), and EBER-ISH(−). Diagnosis: Castleman Disease (Vascular follicular type) (Fig. 1). The patient refused chemotherapy and preferred receive traditional Chinese medicine for treatment. After 9 months, the patient reviewed the cervical lymph nodes, and B-ultrasound showed no significant increase in lymph nodes compared with before. She had no symptoms such as fever, night sweats, or weight loss.

In mid-November 2016, the patient had a cold and developed bilateral nasal obstruction and a runny nose, which was accompanied with a blocked right ear and pain; there was no fever, night sweats, weakness, weight loss, rash or other complaints of discomfort. She visited the local hospital as well as our clinic, and was diagnosed with sinusitis and otitis media. The patient was treated with western medicine (the specific drug is unknown). One month later, her symptoms of obstruction and pain in the right ear were relieved, but the nasal obstruction gradually became intensified. She further complained of significant hyposmia, pain in the left ear and a sensation of a swelling pain around her head, on her forehead, and in other parts of her body. These symptoms were exasperated after feeling cold or tired. For this reason, she visited her local hospital, and her physical examination indicated that “a new neoplasm was visible in the bilateral nostrils”. On December 11, 2016, the patient underwent a puncture through the nasopharynx, for which the pathology indicated non-specific inflammation with lymphoid tissue hyperplasia. Without special handling, she continued to be treated with traditional Chinese medicine.

**Figure 2:**
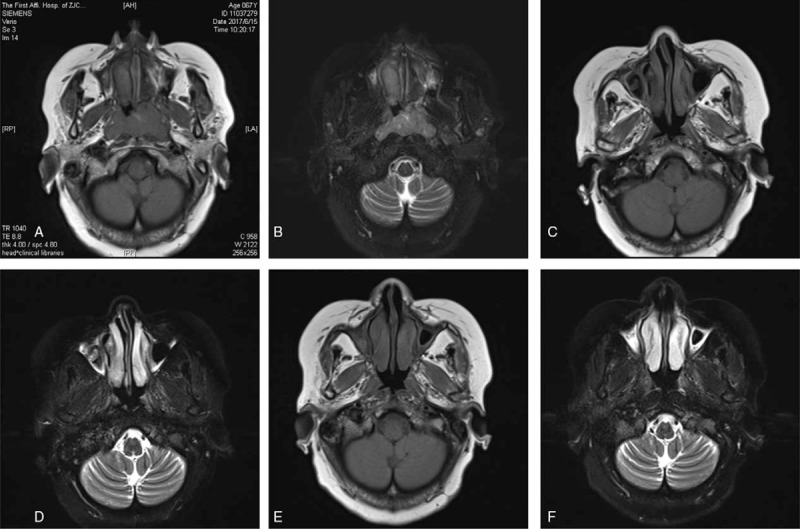
A-B: June 15, 2017 MR showed a Nasopharyngeal soft tissue density, 29 mm × 45 mm. T1WI revealed a low signal, whilst T2WI revealed a high signal. C-D: Before the 4th chemotherapy regimen (October 16, 2017), MR showed that the soft tissue density of the nasopharynx was smaller than before (11 mm × 29 mm); E-F: Before the 5th chemotherapy (December 12, 2017), the MR showed that the size of the nasopharyngeal soft tissue density cavity was 10 mm × 25 mm.

**Figure 3:**
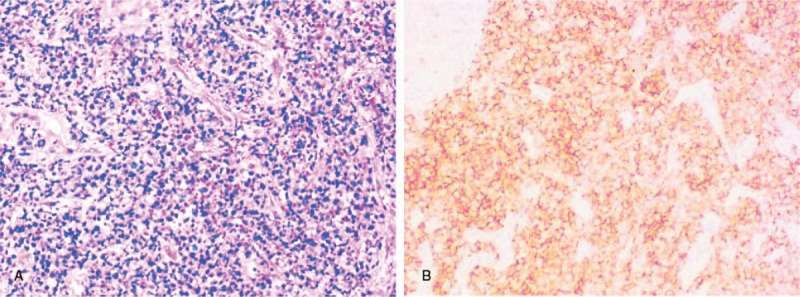
Microscopic histology of PTCL-U: (A) There is a diffuse infiltration of medium-sized abnormal lymphocytes with abundant blood vessels and endothelial cell proliferation with necrosis. (HE × 100); (B) CD3(+).

On June 11, 2017, the patient was admitted to our hospital due to “breathing difficulty and purulent blood in her mouth for more than 20 days”. Physical examination revealed: a large number of neoplasm in both nostrils, which were pale red in color, smooth surfaced, and easy to bleed upon palpation; the left nostril was more evident of this, which more-or-less blocked the nostril. Both sides of the neck, armpit, and groin regions were positively palpated for multiple enlarged lymph nodes. The maximum size was like that of broad beans; they were mobile, soft, smooth surfaced, had clear boundaries, and no significant tenderness. The cardiopulmonary system was unaffected, the liver and spleen were not enlarged, and the neurological examination was unremarkable. Relevant indicators showed that her WBC was 8.0 × 10^9^/L, Hb was 123 g/L, PLT was 223 × 10^9^/L, CRP was 40.39 mg/L; LDH was 171U/L, and EBV-DNA was 7.73E3/ml. Hepatitis-related, HHV-8 and HIV tests were negative. Nasopharyngeal MR revealed a nasopharyngeal space-occupying lesion. The region of the neck close to the carotid artery as well as the neck developed multiple enlarged lymph nodes. Taking into account her medical history, we considered the possibility of a lymphoma. (Fig. 2A-B). Lymph nodes B-ultrasound revealed multiple bilaterally enlarged lymph nodes in the neck, as well as in the supraclavicular, axillary, and groin regions. The largest lesion in the neck was about 2.52 × 1.51 cm; the largest in supraclavicular was about 0.96 × 0.65 cm; the largest in the axillary region was about 2.61 × 0.62 cm; and the largest in the groin was about 2.1 × 0.6 cm. Chest CT plain scan showed a small amount of chronic inflammation scattered throughout the two lungs; the mediastinum was evident for multiple enlarged lymph nodes. Abdominal B-ultrasound and the heart B-ultrasound showed no abnormalities. On June 13, 2017, the patient underwent a biopsy of the nasopharyngeal mass, which revealed (201713580) a “nasopharyngeal” T-cell lymphoma. Immunohistochemistry results: P53(some specific cells+), Ki-67(90%+), EMA(−), CD20(−), CD79a(−), PAX-5(lesions+), Bcl-6(−), MUM1(+), CD2(+), CD3(+), CD4(+), CD5(+), CD7(+), CD8(some+), CD30(+), CD43(+), CD45RO(+), CD56(-), TdT(−), GrB(−), TIA-1(+), ALK(−). Diagnosis considerations: Peripheral T cell lymphoma, unspecified type (PTCL-U) (Fig. 3). No lymphoma cells were found in the bone marrow smear or bone marrow biopsy. Chromosome examination was normal. The final diagnosis: PTCL-U (stage III A, low medium risk); the IPI score was 2 points, and the PIT score was 1 point.

Since June 28th, 2017, the patient received 2 courses of CHOP (Cyclophosphamide 0.8 g qd d1, Adriamycin 40 mg qd d1, Vincristine 3 mg qd d1, Dexamethasone 10 mg d1-5) chemotherapy. The nasopharyngeal obstruction symptoms disappeared completely after chemotherapy. After 2 weeks of rest, the symptoms of nasal obstruction gradually appeared and aggressively progressed. Therefore, the patient was reevaluated before the third cycle of chemotherapy. She was diagnosed with PTCL-U (Stage IIIA, high risk) with an IPI score of 4 points, and a PIT score was 3 points. Since Sep 7th, 2017 to present, the patient was given Chidamide 20 mg/Biw, Sep 7th, 2017 combined with GemOx (Gemcitabine 1.2 g qd d1, Oxaliplatin 100 mg qd d1)+ LASP (Lasparaginase 10,000u qd d6 d8), Oct 23rd 2017 combined with GDP (Gemcitabine 1 g qd, Cisplatin80 mg qd d1, Dexamethasone 40 mg qd d1-d4) and Dec 14th 2017 combined with MA (Methotrexate 1.1 g qd d1, Cytarabine 2.0 g q12 h d2-d3), which helped to completely remove the symptoms of nasopharyngeal blockade. On October 16, 2017 and December 12, 2017, the nasopharyngeal MR revealed that the lesion significantly shrank (Fig. 2C–F) and reached PR. After she was discharged, the patient regularly attended follow-ups and is still alive today.

## 4 Discussion

CD is divided into 3 pathological types in histology: transparent vascular type (HV type), plasma cell type (PC type) and a mixed type (MIX type). According to the extent of lymph node involvement, CD is divided into localized (LCD) and multicentric (MCD), of which more than 90% of LCDs are HV type, while MCDs are mostly PC type and MIX type.^[4]^ The patients in this case had the HV-CD histo-pathological and immunohistochemical characteristics reported in this literature, hence the (cervical) HV-MCD diagnosis was clear. Relevant statistical data showed that the incidence of CD is 21 to 25 cases per million.^[1]^ We searched in medical journal databases using the keywords, “Castleman disease” and “Lymphoma” from 1988 to 2018. We collected almost 120 cases of CD that coexisted with lymphoma (108 cases in Occident, 12 cases in China). In those reports, most CD cases transformed into B-cell lymphomas. Secondary T-cell lymphoma occurred in less than 5% of cases. Only one case, in nearly 20 years, was definitively diagnosed with PTCL-U secondary to CD.^[3]^ In this case, PC-MCD was diagnosed, which transformed into a T-cell malignancy one year later. Subsequently, 14 months later, the patient died due to multiple organ failure caused by severe infection.

The mechanism of CD concurrent to or secondary to a lymphoma is not clear. At present, most studies are considered to be related to chronic viral antigen stimulations, such as HIV, HHV-8, HBV, EBV, which result in over-stimulated normal immune responses, increased IL-6 expression, stimulated abnormal proliferation of B lymphocytes and plasma cells, and increased VEGF expression. In a study that looked at 60 HIV+MCD patients, conducted by Oksenhendler et al^[5]^, 14 patients were developed secondary NHL within 0 to 76 months (median time 20 months); however, baseline variable CD4+ T cell counts and plasma HIV-RNA that were related to HIV-infection could not predict the outcomes of NHL conversion. The Ig or TCR gene rearrangement tests could help determine whether there was clonal hyperplastic lesions or malignant transformation.^[6]^ This patient was negative for HIV, HBV, HHV-8 and ERER-ISH in the peripheral blood, but positive for EBV-DNA.

It is well-known that HHV-8 can encode human homologue IL-6. The proliferation of MCD lymphoid follicles is directly related to IL-6, which is positively correlated with the level of hypersensitive CRP.^[7]^ In this case, HHV-8 test results were negative, so the possibility of HHV-8 infection to secondary lymphoma was excluded. Besides that, CD can also be caused by EB viral infection. It has been reported in the literature that EBER-ISH(+) can be seen in both CD patients and lymphoma patients, and even both can be accompanied by HHV-8(+) and EBV-DNA(+).^[8]^ The cause of PTCL is unknown, but now it has been proven to be related to EBV infection. The mechanism of EBV infection T cells is not clear, and it could be that cytotoxic T cells were infected by EBV when killing EBV infected B cells.^[9]^

When our patient was diagnosed with CD, peripheral blood EBV-DNA was found to be higher than normal despite EBER-ISH being negative. Nasopharyngeal T-cell lymphomas occurred 1.5 years after the diagnosis of HV-MCD. So we suspected that Epstein-Barr virus (EBV) infections may be the common reason behind CD and PTCL-U.

PTCL is a group of highly heterogenic malignant proliferative diseases, of which PTCL-U is the most common type of PTCL. The incidence of PTCL in Europe and the United States accounts for no more than 15% of non-Hodgkin's lymphoma (NHL). The incidence of PTCL in China accounts for about 25% to 30% of NHL. Approximately 70% of patients progressed aggressively, with stage III–IV at the time of their first visits and about half of them experienced systemic symptoms. The IPI/PIT scores mostly indicate a moderate or high risk and are associated with poor prognosis. It has been reported that the median time of progression of PTCL from the time of diagnosis or relapse after remission is 6.7 months, the median PFS is only 3.1 months.^[10]^

The therapeutic effect and prognosis of PTCL-U were related to many factors: clinical stage, PIT score, EBV, Ki-67 and so on. PIT includes 4 risk factors: an age greater than 60; a lactic dehydrogenase (LDH) value at normal levels or above; a performance status (PS) equal to or more than 2; and bone marrow involvement. The PIT score is generally considered to be an independent prognostic risk factor for PTCL-U.^[11]^ Went et al^[12]^ investigated the relationship between 19 kinds of surface markers expressed by PTCL-U and prognosis, which showed that EBER +, CD15 + and Ki-67 ≥ 80% were related to poor prognosis. The clinical significance of Ki-67 ≥ 80% is equivalent to the bone marrow involvement, which indicates a worse chemotherapy response and prognosis. The case was defined as a secondary PTCL-U, the clinical stage was IIIA, and the PIT score was 3 points. Although she had no bone marrow involvement and had a normal karyotype, her Ki-67 reached up to 90%+, which still suggested a poor response to chemotherapy, and a poor prognosis. PTCL-U currently does not have a standard treatment plan, and most patients are not sensitive to classic CHOP or CHOP-like chemotherapy. After this case of PTCL-U emerged in our department, the preferred treatment was 2 courses of CHOP chemotherapy. However, after about half a month of performing undergoing the chemotherapy treatment performed, nasopharyngeal blockage recurred, which suggests that CHOP chemotherapy was ineffective in this patient, and had a poor prognosis. So this patient belonged to relapsed and refractory PTCL-U. Since the use of chidamide 20 mg Biw, combined with GemOx (Gemcitabine, Oxaliplatin) + LASP (LAsparaginase), GDP (Gemcitabine, Cisplatin, Dexamethasone) and MA (Methotrexate, Cytarabine), the symptoms of nasopharyngeal blockade totally disappeared, and the condition was stable. The nasopharyngeal MR lesions were checked again and showed that it reduced by 50%, reached PR.

For relapsed or refractory PTCL, second-line Chemotherapy regimens are generally used, such as GemOx, GDP, or Hyper-CVAD / MA. Related studies have shown that the clinical efficacy of GemOx + asparaginase + dexamethasone Chemotherapy regimen in primary NK / T cell lymphoma patients, and its clinical efficiency can reach up to 91% (62% CR rate, 29% PR rate). Furthermore, a Long-term survival can be obtained (3-year overall survival and disease-free survival were 74% and 57%, respectively).^[13]^ The overall response rate of the GemOx + dexamethasone Chemotherapy regimen, as a salvage regimen for PTCL patients who relapsed or are refractory to other chemotherapy, is 38%.^[14]^ A comparative study that looked at the traditional CHOP or CHOP-like regimen showed that the combined regimen of GemOx + asparaginase had a certain advantage in the treatment of PTCL.^[15]^ More studies have confirmed that the GDP regimen for the treatment of PTCL, especially in stage III-IV patients with moderate to high risk, is significantly more clinically effective than the CHOP regimen, and the incidence of chemotherapy-related toxicity is also lower.^[16]^

As a new subtype, selective histone deacetylase inhibitor (HDACi), Chidamide, is a targeted antitumor drug that is researched independently by China. It selectively inhibits the activity of histone deacetylase (HDAC) in tumor cells, to regulate tumor epigenetics, and to induce and enhance the effect of killing tumor cells; moreover, its selectivity improves tumor cell resistance and inhibits tumor recurrence and metastasis. It has been confirmed that the combination of Chidamide and chemotherapy is particularly suitable for medium and high risk PTCL-U patients, which can significantly improve the chance of remission, and prolonged survival opportunities.^[17]^ With the development and application of new drugs, the patient's survival and quality of life can be further improved.

## Acknowledgments

We thank all our colleagues at the Department of Hematology, The First Affiliated Hospital of Zhejiang Chinese Medical University, and Zhejiang Provincial Hospital of Traditional Chinese Medicine.

## Author contributions

**Conceptualization:** Shuyan Liu.

**Methodology:** Yuanwen Wang, Tonglin Hu.

**Project administration:** Zhiyin Zheng.

**Resources:** Shuyan Liu, Yuanwen Wang, Tonglin Hu, Chunli Zhang.

**Writing – original draft:** Shuyan Liu, Yuanwen Wang.

**Writing – review & editing:** Zhiyin Zheng.
